# Reporting and communication of sample size calculations in adaptive clinical trials: a review of trial protocols and grant applications

**DOI:** 10.1186/s12874-024-02339-7

**Published:** 2024-09-27

**Authors:** Qiang Zhang, Munyaradzi Dimairo, Steven A. Julious, Jen Lewis, Zihang Yu

**Affiliations:** 1 Sheffield Centre for Health and Related Research (SCHARR), School of Medicine and Population Health, University of Sheffield, Sheffield, S1 4DA UK; 2 Department of Biostatistics, University of Washington, Seattle, WA 98195 USA

**Keywords:** Adaptive design, Randomised controlled trial, Sample size estimation, Transparency, Reporting

## Abstract

**Background:**

An adaptive design allows modifying the design based on accumulated data while maintaining trial validity and integrity. The final sample size may be unknown when designing an adaptive trial. It is therefore important to consider what sample size is used in the planning of the study and how that is communicated to add transparency to the understanding of the trial design and facilitate robust planning. In this paper, we reviewed trial protocols and grant applications on the sample size reporting for randomised adaptive trials.

**Method:**

We searched protocols of randomised trials with comparative objectives on ClinicalTrials.gov (01/01/2010 to 31/12/2022). Contemporary eligible grant applications accessed from UK publicly funded researchers were also included. Suitable records of adaptive designs were reviewed, and key information was extracted and descriptively analysed.

**Results:**

We identified 439 records, and 265 trials were eligible. Of these, 164 (61.9%) and 101 (38.1%) were sponsored by industry and public sectors, respectively, with 169 (63.8%) of all trials using a group sequential design although trial adaptations used were diverse.

The maximum and minimum sample sizes were the most reported or directly inferred (*n* = 199, 75.1%). The sample size assuming no adaptation would be triggered was usually set as the estimated target sample size in the protocol. However, of the 152 completed trials, 15 (9.9%) and 33 (21.7%) had their sample size increased or reduced triggered by trial adaptations, respectively.

The sample size calculation process was generally well reported in most cases (*n* = 216, 81.5%); however, the justification for the sample size calculation parameters was missing in 116 (43.8%) trials. Less than half gave sufficient information on the study design operating characteristics (*n* = 119, 44.9%).

**Conclusion:**

Although the reporting of sample sizes varied, the maximum and minimum sample sizes were usually reported. Most of the trials were planned for estimated enrolment assuming no adaptation would be triggered. This is despite the fact a third of reported trials changed their sample size. The sample size calculation was generally well reported, but the justification of sample size calculation parameters and the reporting of the statistical behaviour of the adaptive design could still be improved.

**Supplementary Information:**

The online version contains supplementary material available at 10.1186/s12874-024-02339-7.

## Background

The increasing demands for accelerating the development and testing of healthcare treatments have been noted across sectors [1]. Consequently, the use of adaptive designs (ADs) has had more attention due to their potential to improve efficiency when compared with conventional fixed designs [2, 3], for example, in oncology studies [4, 5]. ADs allow for controlled flexibility to modify design aspects of an ongoing trial based on interim data/results in a manner that preserves the validity and credibility of the trial results [6].

A common theme across ADs is that the trial pathway could change during execution depending on the triggered trial adaptations [7]. There are exceptional circumstances in adaptive trials when there is only one trial path, e.g., in treatment selection adaptation where a rule is to select a fixed number of leading treatments after an interim analysis such as a top-ranked promising treatment. In most cases, the sample size is a variable depending on accumulating trial data and trial path rather than a fixed number of participants known before the start of the trial [8]. For example, in group sequential design, the continuation of the trial depends on the observed data from one or more interim analyses, making the final sample size a discrete variable that can range from the earliest possible stopping point to the latest stage. Therefore, for an AD, it is worthwhile to consider how to report the sample size to reflect the uncertainty in the trial trajectory [9].

Discussion on the reporting of the sample size(s) for ADs matters [10, 11], particularly for trial budgeting and research funding applications. Simply reporting a single sample size in a trial grant application or protocol could be misleading considering the flexibility of ADs as the final sample size has a chance to deviate from the initial reported sample size.

However, despite this, trial registry records (such as on ClinicalTrials.gov) currently require a single specific number for estimated enrolment for all registered trials without considering the implications of trial adaptations.

As the uncertainty in the final trial sample size is contingent upon the types of ADs and the specific decision rules chosen, it is important to establish context-specific minimum requirements when planning sample size(s) in adaptive trials. This is essential for upholding transparency and validity in reporting sample size(s). This transparency, it could be argued, is particularly significant for research grant applications – although this needs to be weighed against restrictions on page or word length.

Existing guidance such as DELTA [12, 13] and the Adaptive designs CONSORT (CONsolidated Standards Of Reporting Trials) Extension (ACE) [6] provide recommendations on what information related to the sample size calculation could be useful when reporting the trial. However, to the best of our knowledge, the question of how to report and communicate the sample size(s) in the trial protocol or grant application for an AD remains unresolved. Given the challenges in reporting trial sample size(s) for an AD, it is beneficial to understand how previous adaptive clinical trials dealt with this problem. This study, therefore, examined previous clinical trials that used ADs to identify good practices and potential gaps for further research.

## Methods

This study was conducted following the methods detailed in the established protocol [14].

### Data sources

We considered trials registered on ClinicalTrials.gov between 01/01/2010 and 12/31/2022. The search also included grant applications submitted within the same period, obtained via email requests from members of the Medical Research Council (MRC)—National Institute of Health and Care Research (NIHR) Trials Methodology Research Partnership (TMRP) Adaptive Designs Working Group (ADWG) (https://www.methodologyhubs.mrc.ac.uk/research/working-groups/adaptivedesigns/) in the UK. This approach enables us to obtain both approved and rejected applications.

To be included in the methodological review, a trial was considered to have used an AD if it was designed with (pre-planned) trial adaptations using interim outcome data/results from participants in the trial. This is consistent with a consensus-driven definition of an AD [15, 16]. A trial was eligible if it:

1. was a phase II, III, or IV clinical trial including those with such seamless components, trials with the phase marked as “non-applicable” or missing were also eligible. This decision was made in recognition that some trials evaluate non-pharmacological interventions (e.g., surgical and behavioural therapies) that may not traditionally fit into a well-defined trial phase typical for pharmacological treatments;
2. was randomised with comparative efficacy or effectiveness objective(s) between treatment arms;
3. had an accessible trial protocol or grant application;
4. used an AD with at least one or more specific types of adaptations (for the definitions of types of ADs considered, refer to Appendix 1).

### Identification of eligible trials

A filter function within the ClinicalTrials.gov registry was used to apply eligibility criteria 1 to 3 above to trial protocols. Following this, a keyword search was conducted on the registry to assess eligibility for criterion 4, using a comprehensive list of search terms (refer to Appendix 2). This search list was initially created based on relevant keywords identified through related literature [17, 18] and discussion with the research team and it was refined through multiple rounds of testing to improve sensitivity and specificity of the search.

Due to the limitations of online searching on ClinicalTrials.gov, which currently does not provide the functionality to search the contents of uploaded documents (such as protocols), an additional searching tool accessible via GitHub was developed by QZ using Python 3.10 (https://github.com/BiosQiang/Comprehensive-searching-on-clinicaltrial.gov).

This complementary search took place after the filtering based on criteria 1 to 3. The eligible trial information was downloaded in batches in XML format. The associated protocols were identified, downloaded and searched using the toolkit. The results of the online search were then combined with the toolkit's findings and duplicate records were removed. Finally, accessed grant applications were integrated with this combined dataset, resulting in the final database for review.

**Fig. 1:**
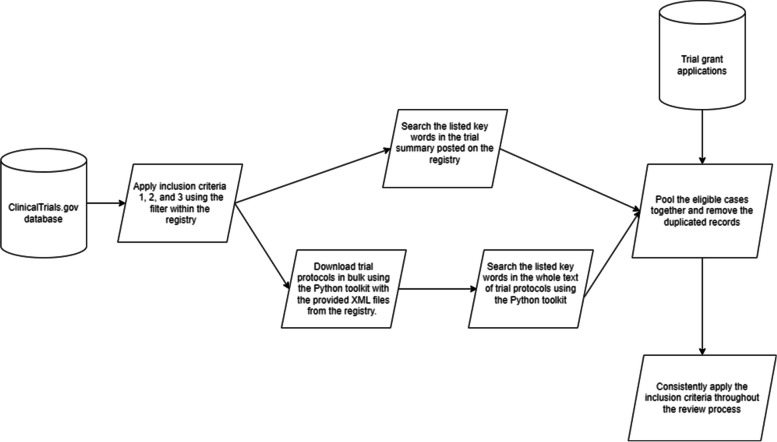
Working flow for the identification of eligible trials

The eligibility criteria were applied continuously throughout the review process to exclude ineligible records. Figure 1 summarises the eligibility screening process.

### Data extraction and analysis

A data extracting sheet was developed to capture key trial information including basic information on trial design; how the sample size was estimated and reported and information on trial adaptations, interim analyses and decision rules. Appendix 3 is a template of the data extraction form.

Two independent reviewers (QZ and ZY) did the data extraction. One reviewer (QZ) searched trials, screening for eligibility, reviewed and extracted required information from all eligible records. The second reviewer (ZY) reviewed 10% of randomly selected records for quality control. If disagreement between two reviewers appeared in less than 20% of all extracted variables, no further independent quality control would be needed as pre-defined in the review protocol [14]. In case of disagreement, MD reviewed the record and then discussed it with QZ and ZY to reach an agreed resolution. Further, quality control was also done through internal data queries by checking for inconsistencies of data extracted between certain variables, e.g., the classed type of AD or adaptive aspects should be consistent with specific trial adaptations. This validation process is recorded in Appendix 4.

The analysis was descriptive using summary statistics depending on the variable type, the distribution of data and the data visualisation approach. The results are reported as guided by the Preferred Reporting Items for Systematic Reviews and Meta-Analyses (PRISMA) 2020 statement [19], where appropriate.

### Example of adaptive design and definitions for technical terms

To facilitate the subsequent discussion, a real trial example with adaptive design is provided in Table 1:

**Table 1:** Example of the ‘ROSE’ study

Reevaluation Of Systemic Early Neuromuscular Blockade study (ROSE, NCT02509078) was a phase III, two-arm, parallel trial testing the efficacy of cisatracurium besylate against a control group in patients with acute respiratory distress syndrome. The primary endpoint was hospital mortality within 90 days of randomization, with a planned sample size of 1,408 participants. Unlike a traditional fixed design, the trial employed a group-sequential design with two evenly spaced interim analyses and one final analysis. The two interim analyses, allowing for the possibility of early trial termination, were planned when 470 and 938 participants had been enrolled. The information time was calculated as the ratio of the 'effective sample size' at each analysis point to the final sample size.The symmetric stopping boundary was designed using the method described by Lan and DeMets [20]	

Reevaluation Of Systemic Early Neuromuscular Blockade study (ROSE, NCT02509078) was a phase III, two-arm, parallel trial testing the efficacy of cisatracurium besylate against a control group in patients with acute respiratory distress syndrome. The primary endpoint was hospital mortality within 90 days of randomization, with a planned sample size of 1,408 participants. Unlike a traditional fixed design, the trial employed a group-sequential design with two evenly spaced interim analyses and one final analysis. The two interim analyses, allowing for the possibility of early trial termination, were planned when 470 and 938 participants had been enrolled. The information time was calculated as the ratio of the 'effective sample size' at each analysis point to the final sample size.The symmetric stopping boundary was designed using the method described by Lan and DeMets [20].

For transparency and to aid interpretation, Table 2 gives some key definitions of technical terms which were used throughout this work:

**Table 2:** Definitions of key technical terms

**Adaptation and type of adaptive design:** according to Chow and Chang [2], an ‘adaptation’ refers to *"a change made to the trial or statistical procedure during a clinical trial."* Adaptive designs are typically categorized based on the types of adaptations implemented. As the design type was often not explicitly defined in original study documents, the author and study team determined design types for each case using a self-developed framework (Appendix 1). This framework is grounded in the consensus of the research team and aligns with classifications found in previous studies [15, 16, 21–26]
**Type of the sponsor:** The sponsor for a clinical trial indicates ‘*An individual, company, institution, or organisation which takes responsibility for the initiation, management, and/or financing of a clinical trial.*’ [27]. According to the type of ownership, a sponsor can be classified as "industry/private sector" or "public" The industry/private sector encompasses businesses that are independently owned and operate for profit without government affiliation. Conversely, the public sector includes organisations owned, controlled and funded by the government or non-profit entities
**Maximum sample size** indicates the possible largest sample size a trial could achieve. For example, in the ROSE study mentioned in Table 1, this would be the sample size of final stage when early stopping is not triggered (1408 participants). In sample size re-estimation designs, it corresponds to the upper limit for sample size increases. In situations where both early stopping and sample size re-estimation are considered as trial adaptations, we use the term "modified maximum sample size" (**mMax**) to refer to the sample size assuming that neither early stopping nor sample size increase is triggered
**Minimum sample size** indicates the theoretically smallest evaluable sample size a trial could achieve. In trials that allow for early stopping, this refers to the sample size on which the earliest interim analysis with the possibility of stopping was planned. For example, in the ROSE study mentioned in Table 1, this would be the number of participants enrolled at the time of the first interim analysis (470 participants)
**Expected sample size** is the long-run average sample size if the same trial was repeated many times under certain scenarios accounting for the fact that considered trial adaptations are triggered in some trials at specific interim analyses. In the ROSE study, this is equivalent to the sum of the stagewise sample sizes weighted by the corresponding probability of stopping at each stage
**Operating characteristics:** entail details of the statistical behaviour of the design in addressing the research question, which can relate to chances of making correct or incorrect decisions under specific scenario(s) [6]. Of note, despite its general recognition that the expected sample size is part of the operating characteristics of the design, the expected sample size is intentionally reported separately in this review

## Results

**Fig. 2:**
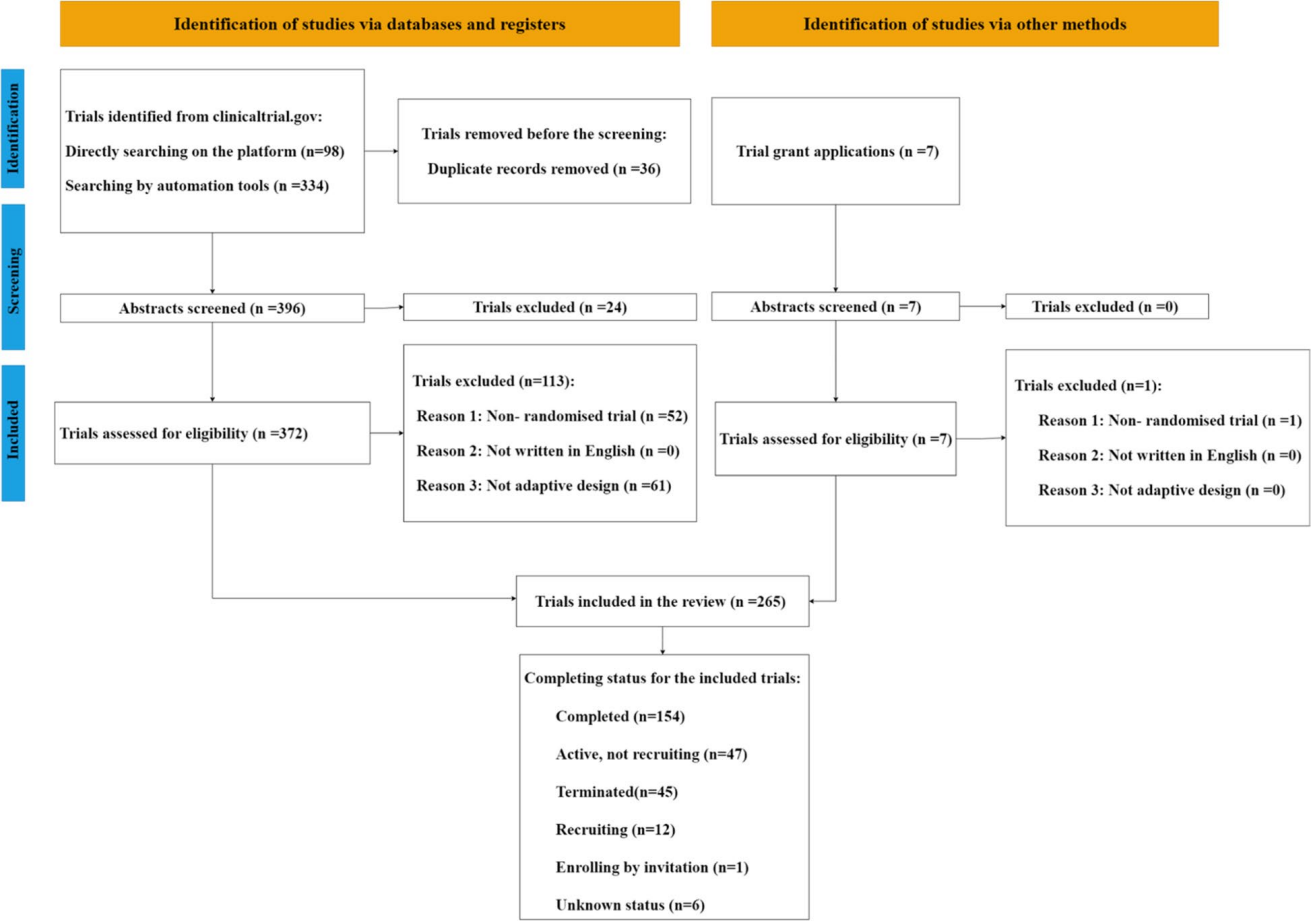
Flowchart for the screening process results

For trial protocols, a total of 432 trials were initially identified through the ClinicalTrials.gov registry (Fig. 2). After removing the duplicates and applying exclusion criteria, 259 eligible records were retrieved. For grant applications, an additional seven trials were obtained and six of these were eligible. Ultimately, 265 eligible trials were reviewed, and of these, 154 (58.1%) had been completed as of the analysis cut-off date (31 December 2022), meaning the last participant's final visit had occurred (including early stopping based on an adaptation), as recorded on ClinicalTrials.gov or reported by the grant application provider.

In addition, since the author of the trial grant application or protocol withheld certain key information upon submission due to confidentiality considerations, the withheld information was marked as 'concealed' in the review results.

### Trial characteristics

As shown in Table 3, among all the 265 eligible trials, 164 (61.9%) and 101 (38.1%) were sponsored by industry (private sector) and the public sector, respectively. Over half, 150 (56.6%), were phase III trials while 38 (14.3%) had their trial phase marked as ‘not applicable’. Over 90% of the industry-sponsored trials evaluated pharmacological treatments (drugs) or drug-involved combinations compared to 69.3% in public sector trials. The proportion of trials with time-to-event and binary primary endpoints was higher in industry and public-sponsored trials, respectively. Moreover, the most common trial design features regardless of the sponsor included superiority primary hypotheses, comparing one treatment against one comparator and use of frequentist methods for the interim and final analyses.

**Table 3:** Design characteristics of eligible trials

Characteristics	Type of sponsor N (%)		Total (*N* = 265)
	**Industry (*N* = 164)**	**Public sector** **(*N* = 101)**	
**Trial phase(s)**			
Phase II	27 (16.5)	25 (24.8)	52 (19.6)
Phase II/III	8 (4.9)	2 (2.0)	10 (3.8)
Phase III	112 (68.3)	38 (37.6)	150 (56.6)
Phase III /IV	1 (0.6)	0 (0)	1 (0.4)
Phase IV	3 (1.8)	11 (10.9)	14 (5.3)
Not applicable^a^	13 (7.9)	25 (24.8)	38 (14.3)
**Class of trial intervention(s)**			
Drug	150 (91.5)	70 (69.3)	220 (83.0)
Device	13 (7.9)	14 (13.9)	27 (10.2)
Clinical procedure	0 (0)	5 (5.0)	5 (1.9)
Surgical treatment	1 (0.6)	4 (4.0)	5 (1.9)
Others^b^	0 (0)	8 (8.0)	8 (3.2)
**Type of primary endpoint(s)**			
Time-to-event	77 (47.0)	21 (20.8)	98 (37.0)
Binary	42 (25.6)	49 (48.5)	91 (34.3)
Continuous	34 (20.7)	25 (24.8)	59 (22.3)
Continuous and binary	5 (3.0)	3 (3.0)	8 (3.0)
Binary and time-to-event	4 (2.4)	3 (3.0)	7 (2.6)
Continuous and time-to-event	2 (1.2)	0 (0)	2 (0.8)
**Type of primary hypothesis test**			
Superiority	149 (90.9)	94 (93.1)	243 (91.7)
Non-inferiority	12 (7.3)	6 (5.9)	18 (6.8)
Superiority and non-inferiority	3 (1.8)	1 (1.0)	4 (1.5)
**Number of study arms**			
Two	131 (79.9)	83 (82.2)	214 (80.8)
Three	22 (13.4)	13 (12.9)	35 (13.2)
More than three arms	11 (6.7)	5 (5.0)	16 (6.0)
**Comparator type**			
Active	91 (55.5)	57 (56.4)	148 (55.8)
Placebo	68 (41.5)	35 (34.7)	103 (38.9)
Standard of care	3 (1.8)	8 (7.9)	11 (4.2)
Active + placebo	2 (1.2)	1 (1.0)	3 (1.1)
**Statistical framework for final analysis**			
Frequentist	157 (95.7)	95 (94.1)	252 (95.1)
Bayesian	5 (3.0)	6 (5.9)	11 (4.2)
Concealed	1 (0.6)	0 (0)	1 (0.4)
Mixed	1 (0.6)	0 (0)	1 (0.4)
**Statistical framework for interim analyses**^**c**^			
Frequentist	151 (92.1)	93 (92.1)	244 (92.1)
Bayesian	6 (3.7)	8 (7.9)	14 (5.3)
Concealed	4 (2.4)	0 (0)	4 (1.5)
Unclear	2 (1.2)	0 (0)	2 (0.8)

^a ^‘non-applicable’ for the trial phase comes from registery information on ClinicalTrials.gov, including trials testing devices, behavioural interventions or other therapies which do not apply to the conventional trial phase. ^b^Including nutrition and behavioural intervention. ^c^Certain cases were marked as ‘unclear’ if the specific method for interim analysis was not described

### Characteristics of adaptive features or trial adaptations

**Fig. 3:**
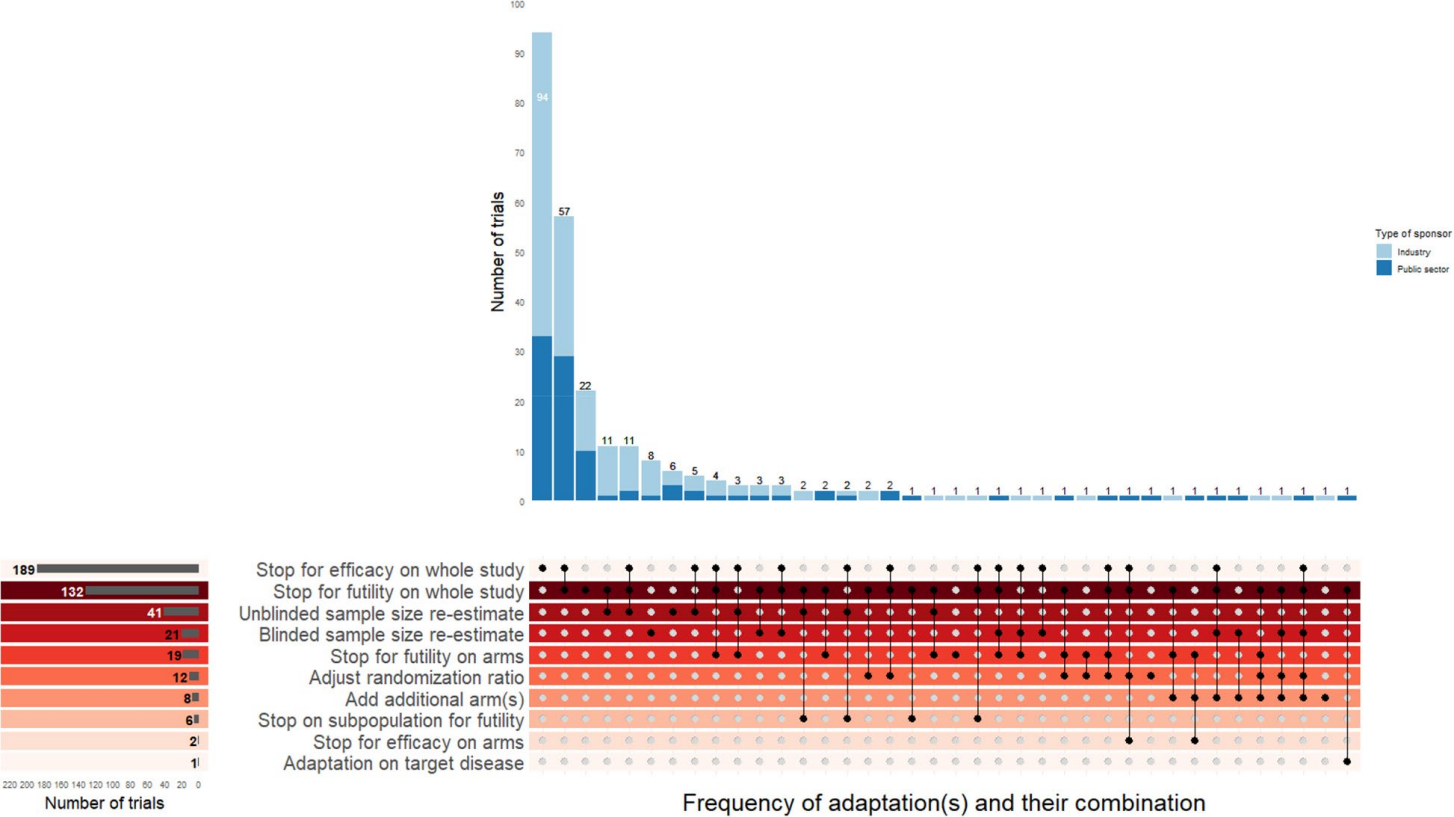
Prevalence of considered trial adaptations and their combinations in reviewed trials. *Figure* 3*is an UpSet plot showing the prevalence of adaptations and their combinations across cases.* *It doesn’t map directly to the adaptive design types in Table* 4., *as the same design can involve different adaptations, and the same adaptation can apply to various* *designs.* *Trials with the details of adaptation concealed or not clearly described were excluded.*

A group sequential design (with options for early stopping the entire trial only) comparing two treatments was the most frequently used type of AD, which accounted for 98 (59.8%) and 71 (70.3%) of industry and public-sponsored trials, respectively (Table 4.). However, trial adaptations considered were diverse (Fig. 3), with early stopping of the entire trial (either for efficacy or futility) as the most common and planned in 238 (89.8%) trials. Multiple trial adaptations were simultaneously considered in a single trial in 61 (23.0%) trials. Sample size re-estimation (including both blinded and unblinded manner) was planned in 63 (23.8%) trials.

**Table 4:** Features of adaptive designs in eligible trials

Features relating to AD	Type of the sponsor N (%)		Total (*N* = 265)
	**Industry** **(*N* = 164)**	**Public sector** **(*N* = 101)**	
**Type of adaptive design**^a^			
Group sequential design	98 (59.8)	71 (70.3)	169 (63.8)
Multiple adaptive design	41 (25.0)	20 (19.8)	61 (23.0)
Sample size re-estimation design	10 (6.1)	3 (3.0)	13 (4.9)
Adaptive treatment selection	3 (1.8)	2 (2.0)	5 (1.9)
Adaptive seamless design	3 (1.8)	1 (1.0)	4 (1.5)
Adaptive basket design	0 (0)	1 (1.0)	1 (0.4)
Adaptive enrichment design	0 (0)	1 (1.0)	1 (0.4)
Adaptive hypothesis design	1 (0.6)	0 (0)	1 (0.4)
Response adaptive randomisation	0 (0)	1 (1.0)	1 (0.4)
Concealed	8 (4.9)	1 (1.0)	9 (3.4)
**Adequately described the interim decision rule(s)**^**b**^			
Yes	141 (86.0)	88 (87.1)	229 (86.4)
Partially	4 (2.4)	6 (5.9)	10 (3.8)
No	11 (6.7)	5 (5.0)	16 (6.0)
Concealed	8 (4.9)	2 (2.0)	10 (3.8)
**Number of pre-planned interim analyses**			
One	111 (67.7)	51 (50.5)	162 (61.1)
Two	33 (20.1)	20 (19.8)	53 (20.0)
Three	5 (3.0)	10 (9.9)	15 (5.7)
Four or more	5 (3.0)	11 (10.9)	16 (6.0)
Not stated	2 (1.2)	8 (7.9)	10 (3.8)
Concealed	8 (4.9)	1 (1.0)	9 (3.4)
**Was there any pre-planned early stopping**			
Yes	144 (87.8)	94 (93.1)	238 (89.8)
No	13 (7.9)	6 (5.9)	19 (7.2)
Concealed	7 (4.3)	1 (1.0)	8 (3.0)
**Type of early stopping considered on the whole study**	(*n* = 144)	(*n* = 94)	(*n* = 238)
Efficacy	69 (47.9)	37 (39.4)	106 (44.5)
Efficacy and futility	43 (29.5)	40 (42.6)	83 (34.9)
Futility	32 (22.2)	17 (17.9)	49 (20.6)
**Type of the futility stopping rule considered on the whole study** ^c^	(*n* = 75)	(*n* = 57)	(*n* = 132)
Non-binding	32 (41.0)	5 (8.5)	37 (27.0)
Binding	7 (9.0)	9 (15.3)	16 (11.7)
Not stated	36 (48)	43 (75.4)	79 (59.8)
**Planned sample size re-estimation**			
Yes	47 (28.7)	16 (15.8)	63 (23.8)
No	117 (71.3)	85 (84.2)	202 (76.2)
**Method for sample size re-estimation**^d^	(*n* = 47)	(*n* = 16)	(*n* = 63)
Unblinded comparative	28 (59.6)	8 (50)	36 (57.1)
Blinded non-comparative	14 (29.8)	6 (37.5)	20 (31.7)
Unclear^e^	4 (8.5)	1 (6.2)	5 (7.9)
Concealed	1 (2.1)	0	1 (1.6)
Unblinded non-comparative	0	1 (6.2)	1 (1.6)
**Information used for sample size re-estimation**	(*n* = 47)	(*n* = 16)	(*n* = 63)
Nuisance parameters	14 (29.8)	7 (43.8)	21 (33.3)
Interim treatment effect	28 (59.6)	8 (50)	36 (57.1)
Not stated	4 (8.5)	1 (6.2)	5 (7.9)
Concealed	1 (2.1)	1 (2.1)	1 (2.1)

^a^The type of AD was classed based on the definitions given in Appendix 1, and further classification of ‘multiple adaptive design’ according to its main objective is given in Appendix 5. ^b^Indicates whether the content of the trial adaptation(s) and the conditions to trigger corresponding adaptation described. ^c^The denominator is the number of trials with at least a pre-planned futility early stopping rule. ^d^The denominator is the number of trials with pre-planned sample size reestimation. ^e^The method of sample size re-estimation was marked as ‘unclear’ if the method used for re-estimation was not clearly described

**Fig. 4:**
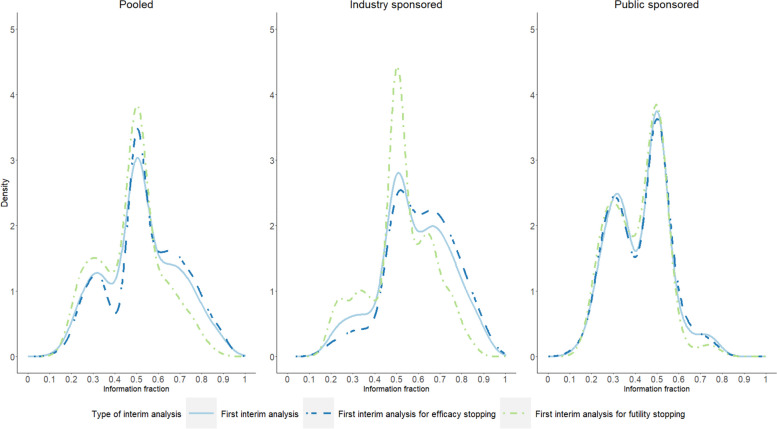
Distribution of the information fraction of the first interim analysis

Having one interim analysis (*n* = 162, 61.1%) and the first interim analysis planned at the halfway point of the trial were the most common interim decision-making features (Fig. 4). Public-sponsored trials tended to have more interim analyses compared to industry-sponsored trials (Table 4.). Some public-sponsored trials planned the first interim analysis earlier, at one-third of the trial, as evident by the bimodal distribution in the timing of analysis (Fig. 4). The proportion of trials with pre-planned sample size re-estimation was higher in industry-sponsored cases (*n* = 48, 29.3% vs *n* = 17, 16.8%) and the industry-sponsored trials were more likely to perform this sample size re-estimation in an unblinded-comparative way.

Although most of the trials (*n* = 229, 86.4%) were able to clearly describe the interim decision rule(s), not all of them are replicable, e.g., 84 (61.3%) trials did not state whether the futility stopping rule would be binding or non-binding.

### Sample size reporting methods and the estimated enrolment

Table 5. summarises the reporting of sample size aspects. Most sample size calculations (*n* = 229, 86.4%) were performed analytically and 216 (81.5%) trials described the sample size calculation methods used in detail making them reproducible; the reproducibility rate is higher in group sequential designs (147, 87.0%) and lower in multiple adaptive designs (48, 78.7%) and adaptive treatment selection designs (3, 60.0%) (Appendix 6). For the general items related to sample size that apply for both fixed and ADs, more than two-thirds of the trials regardless of sponsor did not disclose the statistical software, package, or code used for the calculation. Moreover, there was no justification for the choice of parameters used for sample size calculation in 79 (48.2%) and 37 (36.5%) industry-sponsored and public-sponsored trials, respectively.

**Table 5:** Reporting of sample size aspects

Sample size aspect	Type of sponsor N (%)		Total (*N* = 265)
	**Industry** **(*N* = 164)**	**Public sector** **(*N* = 101)**	
**Method for sample size calculation**			
Analytical	144 (87.8)	85 (84.2)	229 (86.4)
Simulation	8 (4.9)	11 (10.9)	19 (7.2)
Not stated	2 (1.2)	4 (4.0)	6 (2.3)
Concealed	10 (6.1)	1 (1.0)	11 (4.2)
**Statistical software used for sample size calculation**			
Software and name of package/macro/function were described	43 (26.2)	18 (17.8)	61 (23.0)
Only the name of the software	6 (3.7)	3 (3.0)	9 (3.4)
Not stated	100 (61.0)	80 (79.2)	180 (67.9)
Could not be ascertained^a^	15 (9.1)	0 (0)	15 (5.7)
**Sample size calculation well-described to be reproducible**			
Yes	133 (81.1)	83 (82.2)	216 (81.5)
No	20 (12.2)	17 (16.8)	37 (14.0)
Concealed	11 (6.7)	1 (1.0)	12 (4.5)
**Were the parameters well stated?**			
Yes	143 (87.2)	91 (90.1)	234 (88.3)
Partially	0 (0)	1 (1.0)	1 (0.4)
No	10 (6.1)	8 (7.9)	18 (6.8)
Concealed	11 (6.7)	1 (1.0)	12 (4.5)
**Was the choice for parameters justified?**			
Yes	74 (45.1)	63 (62.4)	137 (51.7)
No	79 (48.2)	37 (36.6)	116 (43.8)
Concealed	11 (6.7)	1 (1.0)	12 (4.5)
**Which part of the justification for parameters was missing?**^b^	(*n* = 79)	(*n* = 37)	(*n* = 116)
Effect size and nuisance parameters	58 (73.4)	32 (86.5)	90 (77.6)
Effect size	21 (26.6)	5 (13.5)	26 (22.4)
**Was the minimum sample size reported?**			
Yes	150 (91.5)	86 (85.1)	236 (89.1)
No	6 (3.7)	14 (13.9)	20 (7.5)
Concealed	8 (4.9)	1 (1.0)	9 (3.4)
**How was the minimum sample size reported?**	(*n* = 150)	(*n* = 86)	(*n* = 236)
Stated	10 (6.7)	6 (7.0)	16 (6.8)
Inferred by the interim analyses plan and maximum sample size	140 (93.3)	80 (93.0)	220 (93.2)
**Was the maximum sample size reported?**			
Yes	144 (87.8)	95 (94.1)	239 (90.2)
No	13 (7.9)	6 (5.9)	19 (7.2)
Concealed	7 (4.3)	0 (0)	7 (2.6)
**Was the expected sample size reported?**			
Yes	4 (2.4)	14 (13.9)	18 (6.8)
No	151 (92.1)	86 (85.1)	237 (89.4)
Concealed	9 (5.5)	1 (1.0)	10 (3.8)
**Under what scenario(s) was the expected sample size calculated**	(*n* = 4)	(*n* = 14)	(*n* = 18)
H_0_ and H_1_	3 (75.0)	6 (42.9)	9 (50.0)
H_0_, H_1_ and other assumption(s)^c^	1 (25.0)	6 (42.9)	7 (38.9)
H_1_	0 (0)	2 (14.3)	2 (11.1)
**What was the planned enrolment**			
Maximum	106 (64.6)	79 (78.2)	185 (69.8)
Maximum (mMax)^d^	41 (25)	15 (14.9)	56 (21.1)
Minimum	14 (8.5)	2 (2.0)	16 (6.0)
Not stated	3 (1.8)	5 (5.0)	8 (3.0)

^a^The reporting for tools for sample size calculation could not be ascertained when the sample size calculation part in the protocol was concealed. ^b^Only for trials without parameters justification, the proportion is calculated accordingly. ^c^Indicates other assumed effect sizes which were believed to be possible or had been observed in previous studies. ^d^As defined in Table 2

For items specific to ADs, the maximum, minimum, or expected sample size were metrics reported in most of reviewed trials. The maximum sample size was the most frequently reported. The minimum sample size was directly stated only in 6.0% of trials (16/265), but it was able to be directly inferred from information provided on the interim analyses plan in 220 (92.3%) trials.

**Fig. 5:**
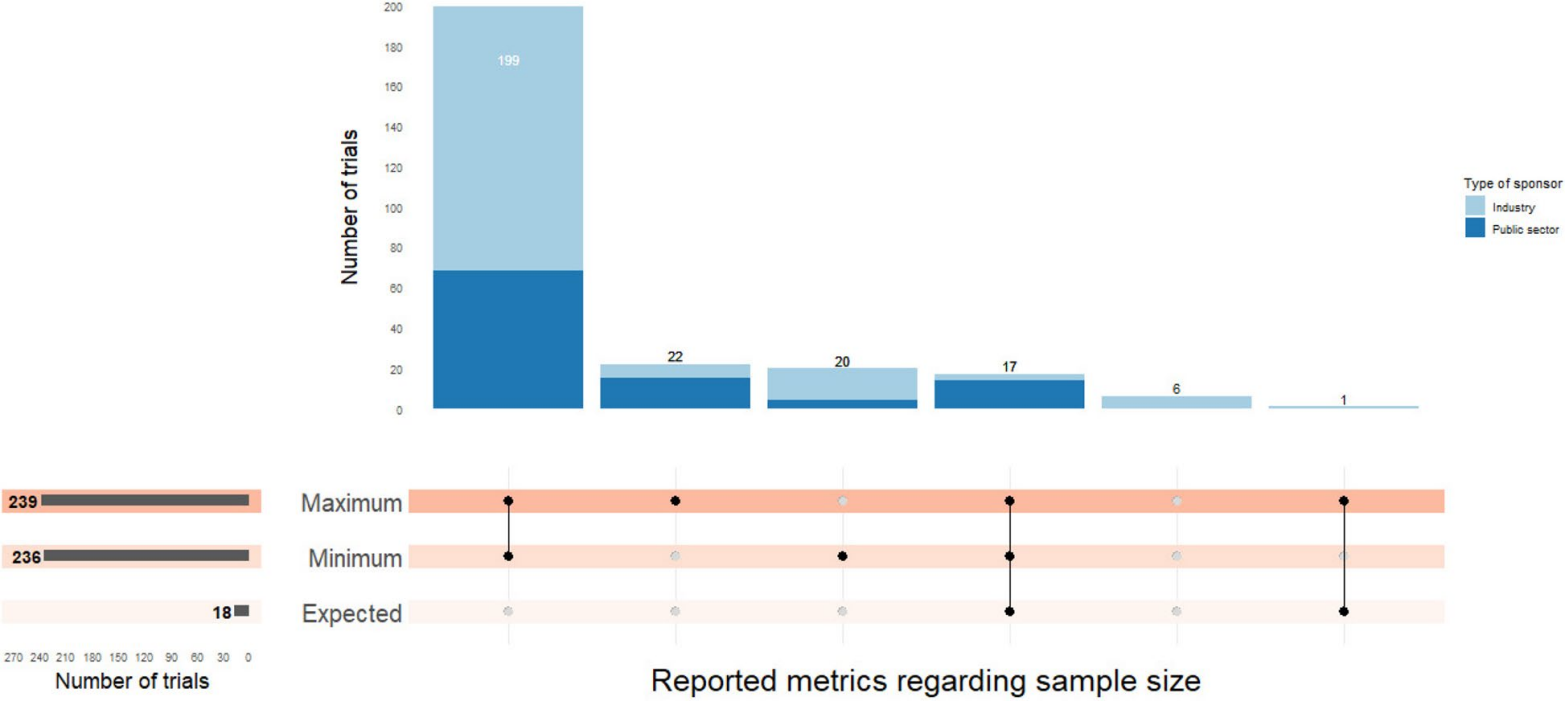
Reporting of sample size quantities (excluding 7 trials with sample size concealed)

Reporting the maximum and minimum sample size simultaneously was most common (Fig. 5). In contrast, only 18 (6.8%) trials reported the expected sample size, of which, 9 (50.0%) were calculated under both the null and alternative hypotheses (Table 5.).

For the enrolment of an adaptive design, the plan could be based on the minimum, maximum, or modified maximum (mMax, as defined in the methods section). Table 6 presents the metrics used by different adaptive designs to estimate enrolment. Typically, the planned metric is determined based on the sample size under the assumption that no adaptations will be triggered: for trials with early stopping as the only adaptation (e.g. group sequential design), this would be the maximum sample size; for trials with sample size re-estimation as the only adaptation, it would be the minimum sample size. When early stopping and sample size re-estimation were both planned, the target sample size was usually set assuming neither early stopping nor sample size increase would take place.

**Table 6:** Enrolment Estimation Across Adaptive Design Types

Type of adaptive design	Metrics for enrolment estimation			
	**Maximum**	**Minimum**	**mMax**^**a**^	**Not stated**
Group-sequential design (*n* = 169)	169 (100.0)	0 (0)	0 (0)	0 (0)
Multiple adaptive design (*n* = 61)	8 (13.1)	2 (3.3)	50 (82.0)	1 (1.6)
Sample size re-estimation design (*n* = 13)	0 (0)	12 (92.3)	0 (0)	1 (7.0)
Adaptive treatment selection (*n* = 5)	2 (40.0)	1 (20.0)	0 (0)	2 (40.0)
Adaptive seamless design (*n* = 4)	4 (100.0)	0 (0)	0 (0)	0 (0)
Adaptive basket design (*n* = 1)	0 (0)	0 (0)	0 (0)	1 (100.0)
Adaptive hypothesis design (*n* = 1)	0 (0)	1 (100.0)	0 (0)	0 (0)
Adaptive enrichment design (*n* = 1)	1 (100.0)	0 (0)	0 (0)	0 (0)
Response adaptive randomisation (*n* = 1)	1 (100.0)	0 (0)	0 (0)	0 (0)

^a^mMax indicates modified maximum sample size, as defined in Table 2

#### Reporting of the operating characteristics for the adaptive design

Table 7 summarises the details of operating characteristics of the AD that were explicitly stated. Less than half of the trials provided information on the operating characteristics of the AD. Among all the reported terms, the stagewise type I error rate and cumulative power at the interim analysis were the most frequently reported terms while relatively fewer trials had reported the probability for early stopping or taking certain trial adaptations.

**Table 7:** Reporting of operating characteristics

Reporting of operating characteristics	Type of sponsor N (%)		Total (*N* = 265)
	**Industry (*N* = 164)**	**Public sector (*N* = 101)**	
**Were the statistical operating characteristics of the design reported?**^**a**^			
Yes	72 (43.9)	47 (46.5)	119 (44.9)
No	80 (48.8)	53 (52.5)	133 (50.2)
Concealed	12 (7.3)	1 (1.0)	13 (4.9)
**Stagewise type I error (false positive rate) rate at an interim analysis**^b^	(*n* = 72)	(*n* = 47)	(*n* = 119)
Yes	70 (97.2)	43 (91.5)	113 (95.0)
No	2 (2.8)	4 (8.5)	6 (5.0)
**Cumulative statistical power**^b^	(*n* = 72)	(*n* = 47)	(*n* = 119)
Yes	62 (86.1)	45 (95.7)	107 (89.9)
No	10 (13.9)	2 (4.3)	12 (10.1)
**Probability of early efficacy stopping on the entire trial**^c^	(*n* = 67)	(*n* = 41)	(*n* = 108)
Yes	8 (11.9)	6 (14.6)	14 (13.0)
No	59 (88.1)	35 (85.4)	94 (87.0)
**Probability of early futility stopping on the entire trial**^c^	(*n* = 33)	(*n* = 26)	(*n* = 59)
Yes	7 (21.2)	5 (19.2)	12 (20.3)
No	26 (78.8)	21 (80.8)	47 (79.7)
**Probabilities for triggering adaptations**^**d**^	(*n* = 5)	(*n* = 4)	(*n* = 9)
Yes	3 (60.0)	1 (25.0)	4 (44.4)
No	2 (40.0)	3 (75.0)	5 (55.6)

^a^The operating characteristics will be regarded as having been reported if at least one of the followings was involved: the stagewise type I error rate (false positive rate), cumulative statistical power, the probability of early trial termination at each interim analysis and the probabilities of triggering other adaptations. ^b^The denominator was the number of trials that have reported the corresponding operating characteristics. ^c^Only applicable for trials with related trial adaptations. ^d^The denominator is the number of trials with planned adaptations for which it is possible to give the triggering probability under a certain scenario (except early stopping entire trials on efficacy or futility). For the reviewed trials,the reported probabilities include that of dropping a certain arm, stopping a sub-population and expanding to the next clinical study phase

### Comparison between the actual enrolment and planned enrolment size

**Fig. 6:**
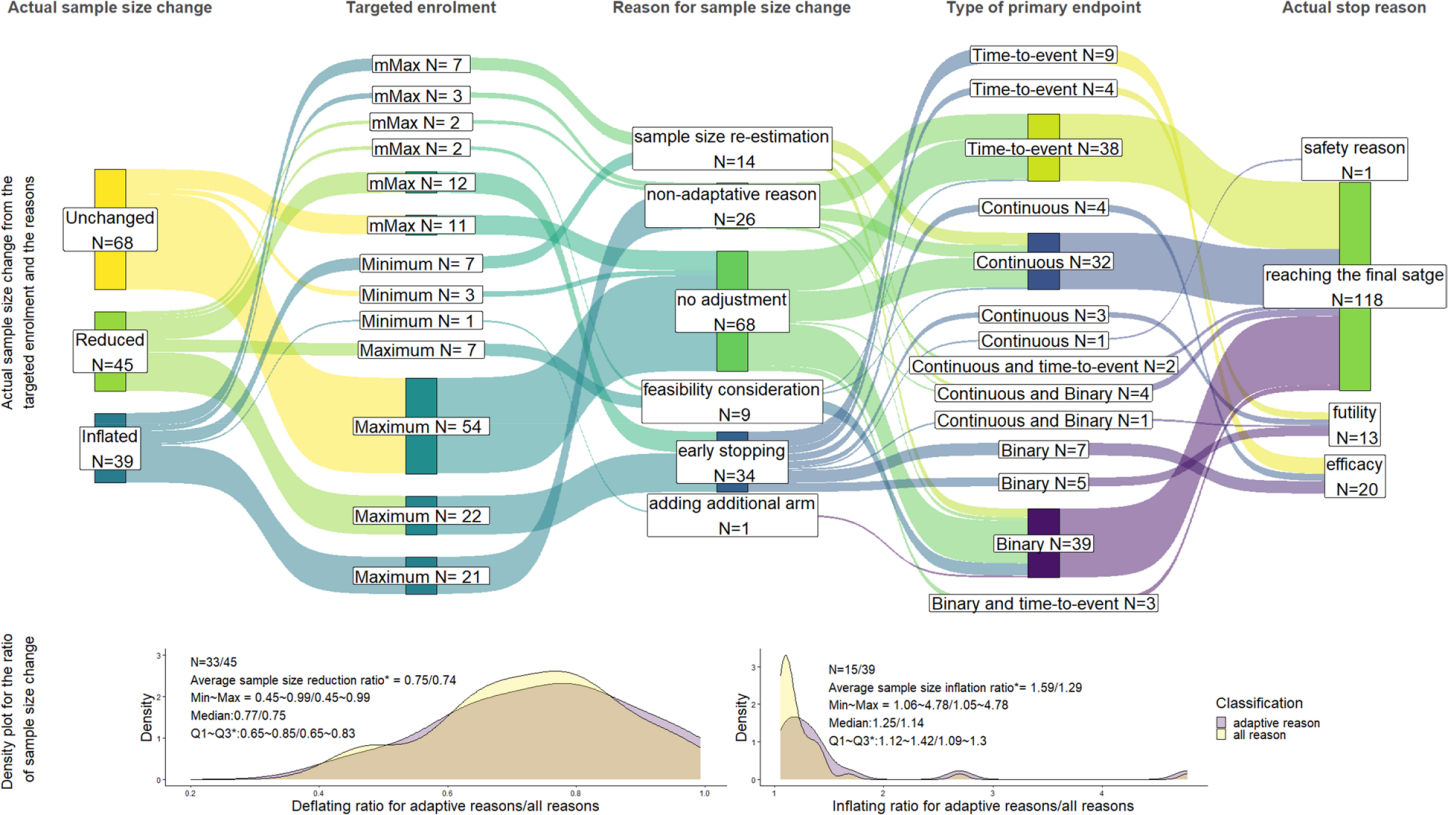
Sankey plot for the actual enrolment status among all completed trials. *The number before and after the ‘/’ corresponds to change due to adaptive reasons and all reasons respectively. Q1 ~ Q3: The first ~ third quartile

Figure 6 illustrates how the sample size changed from the original estimation and the corresponding reasons using a Sankey plot. Additionally, the scale of the change is depicted in the accompanying density plot. Of 154 completed trials, 152 (98.7%) had information on their actual enrolment available. Of these 152 trials, 68 (44.7%) enrolled the planned sample size without significant deviation (unless modification on the sample size was specifically stated in the study results, any deviation above 5% of the original sample size was deemed significant), while 45(29.6%) had fewer participants than planned and 39 (25.7%) had more participants than planned (Fig. 6).

Of the completed trials with inflated sample size, 15/39 (38.5%) trials were a result of trial adaptations, with the majority of these triggered by sample size re-estimation (*n* = 14) and only one trial due to the addition of a new treatment arm. The remaining 24/39 (61.5%) trials had an inflated sample size for reasons not related to trial adaptations considered (e.g., increasing the sample size to compensate for a lower event rate than expected); of which, 16 (66.7%) trials had a time-to-event primary endpoint. As a result of taking adaptations, the average ratio for sample size increasing was around 1.6.

With respect to trials that had a reduced sample size at completion, 33 /45 (73.3%) were due to pre-planned early stopping (*n* = 13, 28.9% and *n* = 20, 44.4% for futility and efficacy, respectively), while 7 trials stated that the sample size was decreased due to feasibility considerations and 1 trial was terminated early due to safety considerations. The average reduction in sample size for adaptive reason was 25%.

### Examples of reporting sample size calculation for an adaptive trial

Regarding the ‘ROSE’ study (NCT02509078) introduced in Table 1, it was able to provide some information regarding the sample size and trial operating characteristics in the protocol.

In terms of the sample size estimation, nuisance parameters, targeted effect size and the corresponding justification were all provided (Table 8).

*“The presumed 35% mortality rate in the control group is based on several recently published clinical trials. […] The table below shows the effect of changes in the power of the study as a function of the mortality rate on the treatment. We calculated the power under two assumptions. The first is that the absolute difference in mortality rates was 8% and the second was that the relative difference was 23% which is approximately 8% of the anticipated mortality of 35%. The second row of the table shows the current assumptions. Whether you fix the absolute or the relative difference, the power is above 80% as long as the null mortality rate is over 25%. The power goes down to 73% if the mortality rate is 20% which is below the mortality rate observed in a ARDSNet (*Acute Respiratory Distress Syndrome Network*) study.”*

**Table 8:** Change in power against different effect size assumptions (recreated from ROSE protocol)

Null mortality	Power at 8% absolute decrease in mortality	Power at 23% relative decrease in mortality
45%	86%	97%
35% ^a^	90%	90%
30%	93%	85%
25%	96%	79%
20%	98%	73%

^a^Assumption of the best estimate of control event rate used in the ROSE Protocol

Regarding the adaptation, it states:

*“There will be two interim analyses and a final analysis conducted when approximately each successive 1/3 of the patients have been enrolled. […] This trial will stop for superiority of either active or control and is designed with symmetric group sequential flexible stopping boundaries as described in Lan and DeMets (with corresponding reference),* […] *The information time was calculated as the ratio of the 'effective sample size' at time of each look to the final sample size.”*

Regarding the enrolment, it states:

*“The maximum required total sample size is 1408 subjects. […] The trial will accrue a maximum of 1408 patients.”*

As shown in Table 9., the trial also provided detailed information on the decision rules and operating characteristics.

*“The table presents stopping boundaries as both a required observed mortality difference and a one-sided p-value. The columns under ‘Probability of Stopping’ present the probability of stopping at each stage under the null and alternative.”*

**Table 9:** Information on planned interim analysis and the stopping probabilities (recreated from ROSE protocol)

Number of subjects	Active superior		Control superior		Probability of stopping	
	**Mortality difference**	***P*-value**^**a**^	**Mortality difference**	***P*-value**^**a**^	**Under H0**^**b**^	**Under H1**^**b**^
470	-0.146	0.00031	0.146	0.99969	0.001	0.061
938	-0.078	0.00479	0.078	0.99521	0.010	0.528
1408	-0.049	0.02361	0.049	0.97639	0.050	0.900

^a^These are one-sided p values for the upper and lower boundaries

^b^H0 and H1 mean the null and alternative hypotheses, respectively

Although the minimum and the expected sample sizes were not explicitly stated, they can be worked out from the information provided.

## Discussion

Accurately reporting the sample size can help readers understand the study design and ensures the reproducibility of methods [28]. It is also important for adequate planning of the trial budget [29], especially when an AD is used [30], since triggered trial adaptations can lead to different sample sizes.

Regarding the metrics of the sample size, the minimum and maximum values establish the range of anticipated information as well as the budgetary limitations in trial execution, which may usually make the reporting of these two metrics in trial plans reasonable. This is consistent with extant literature [31]. However, merely giving these ‘extreme’ sample size(s) might be insufficient since the minimum or maximum sample size is not always the one to be eventually achieved.

Previous studies indicated that the trial budget for an AD was usually estimated assuming the ‘worst’ situation [31] among all possible trial trajectories. This is true when early stopping is the only adaptation. Our review confirms this finding and extends it to: most trials were found to use the sample size assuming no adaptation would be triggered for planning purposes: with no sample size re-estimation, this would be equivalent to the maximum sample size; when sample size re-estimation is the only adaptation, this would correspond to the original sample size which is also the minimum.

Using the sample size assuming there will be no trial adaption in the trial plan provides a simple solution regarding the sample size to use when designing a trial, but it is not always the best choice. In this review, 48/152 (31.6%) of completed trials had their sample size changed from the original enrolment estimation for trial adaptation reasons. Considering this, reporting additional information on the sample size and planned trial adaptation could potentially reduce the allocated trial budget, if the trial is very likely to stop early [10, 32–34].

For an AD, other metrics, such as the most likely sample size (in terms of probability), or the expected sample size, may convey useful information to review of the grant applications and protocols and may be more meaningful on the trial portfolio level. The reporting of these metrics relies on transparent presentation of the operating characteristics of the AD, especially the probability for triggering an adaptation and when interim analysis will be performed. Gsponer et al. state that for trials with planned early stopping, the probability of stopping for success, futility and the likelihood of continuing at each interim analysis should be the main interest when giving the operating characteristics [34]. This information is of particular importance when communicating the design to funders [30] and other trial stakeholders (e.g., regulators).

Thus, it would be valuable to discuss in further studies on how to report the operating characteristics of adaptive designs and how they should be applied across different scenarios, including different statistical frameworks (frequentist and Bayesian) and complex design types such as biomarker enrichment design [35].

Our review found that the type of sponsor of the trial could impact design features and thus could possibly affect the sample size determination as well as its reporting: for example, publicly sponsored trials had their first analysis earlier than industry-sponsored trials with the time of the first analysis being bimodally distributed. One possible explanation could be that public sponsored being more sensitive to budget limitations—which may make terminating futile or unfeasible studies—in terms of ability to recruit – more desirable. It has been reported that almost all UK-based public trials from 2012 to 2016 had an internal pilot to assess feasibility in recruitment (i.e., if the study was on track to reach recruitment targets) [36]. Of these internal pilots, two-thirds were done typically after 20% of recruitment. It could be that if the internal pilot was being undertaken anyway that a study team also undertakes a formal interim analysis for futility – which could probably explain the first peak at 30% of information fraction during interim analysis, assuming prompt information of endpoint on recruitment. The gap between 20 and 30% possibly being due to aiming for more information to form an adaptive decision [37]. In this context, always allocating the budget by assuming no adaptation would take place might be questionable for certain trials (e.g., when aggressive stopping rules are used) or may not be the most effective solution from a portfolio perspective.

Finally, the different roles of researchers in clinical trial research can also influence their focus on different metrics of sample size. For public sponsors, knowing the maximum sample size helps decide the feasibility of recruitment, which is recognized as the most difficult area for determining the success of a trial [38]. For industry sponsors, knowing the minimum sample size, especially in pivotal studies, determines the least statistical information that should be gathered to support regulatory submissions for a study treatment. For both public and industry sponsors knowing the expected sample size can help allocate funding.

## Conclusion

The reporting of sample size(s) for ADs demands more careful considerations than that of conventional fixed designs. Reporting the maximum, minimum and expected sample size as well as the operating characteristics in trial grant applications and protocols can provide valuable information that can be used for different purposes by diverse stakeholders.

The current review has revealed that the maximum and minimum sample sizes were commonly reported and the sample size assuming no adaptations were triggered is usually set as the target enrolment. This is despite the review showing that the actual enrolment and the target sample size did differ due to triggered trial adaptations.

The sample size calculations, details of trial adaptations and the interim decisions were generally well reported. However, this may not be generalisable to adaptive trials without accessible protocols and there is still room for improvement in justifying the parameters selected to calculate sample size and reporting the statistical behaviour of the design.

Since there are no recommendations on best practice for reporting of sample sizes for ADs in grant applications and protocols, this work has shown that further work is required to provide structured practical guidance.

## Supplementary Information

Supplementary Material 1.

## Data Availability

The protocol and audit data associated with this study, along with the corresponding source code for comprehensive searching toolkit, are available for access and verification. The protocol and audit data are deposited in the Open Research Data Archive (ORDA), ensuring their accessibility for interested parties. Additionally, the source code for the searching toolkit is hosted on GitHub, providing a transparent and collaborative platform for the examination and utilization of the codebase. Researchers and stakeholders interested in accessing these resources can find the protocol and audit data on ORDA (https://figshare.shef.ac.uk/authors/Qiang_Zhang/11637799) and the source code for the searching toolkit on GitHub (https://github.com/BiosQiang/Comprehensive-searching-on-clinicaltrial.gov).

## Abbreviations

AD(s): Adaptive design(s)
ADWG: Adaptive designs working group
CONSORT: Consolidated standards of reporting trials
mMax: Modified maximum sample size (see definition provided in Table 2)
MRC: Medical research council
NIHR: National institute of health and care research
PRISMA: Preferred reporting items for systematic reviews and meta-analyses
TMRP: Trials methodology research partnership
