# Supplementary material for: Reporting and communication of sample size calculations in adaptive clinical trials: a review of trial protocols and grant applications
Source: BMC Med Res Methodol. 2024 Sep 27;24:216. doi: 10.1186/s12874-024-02339-7 (PMC11430544; doi:10.1186/s12874-024-02339-7)
Supplement: Supplementary file 1 — Supplementary Material 1. [file 12874_2024_2339_MOESM1_ESM.docx]

# Appendix

**Appendix 1 The classification rule of AD types**

| **Name of AD** | **Description of the classification rule** |
| --- | --- |
| Group sequential design | A design sequentially enrols participants and at an interim analysis, a study treatment is compared to a comparator based on accrued outcome data from a group of participants to that point and a decision is made on whether to stop the trial early because there is sufficient evidence to conclude (e.g., futility or efficacy) while accounting for repeated significance testing. This is often referred to as a standard group sequential design, we used this definition to minimize the overlap with other design types, especially multi-arm multi-stage design (see below). |
| Sample size re-estimation design | A design with options to change (increase or decrease) the initial sample size by re-estimating sample size parameters at an interim analysis. This can be done in a comparative or non-comparative manner [22]. |
|  |  |
| Response adaptive randomisation | A design that allows changes to how participants are allocated to treatments by giving more weight to study treatments that are showing promising benefits based on interim results. A statistical method is used to weight interim results to update randomisation ratios. This excludes any changes to randomisation ratios independent of the interim results (e.g., based on baseline characteristics of participants [23]. |
|  |  |
| Adaptive enrichment design | A design that allows modifying the characteristics of participants recruited into a trial to focus on those participants that are more likely to benefit from study treatment based on interim results. That is, after an interim analysis, the target population may remain the same as the originally planned or narrowed to focus on a subset of participants with specific characteristics (subpopulation) |
|  |  |
| Adaptive hypothesis design | A design that allows modifying the hypothesis (e.g., from non-inferiority to superiority) based on the interim analysis results. |
|  |  |
| Adaptive platform design | A design that studies multiple interventions’ effects on a condition or disease within a single trial, under a master (core) protocol; during the trial process, adaptations such as dropping/adding arm (s) or switching the control arm were allowed [24]. |
| Adaptive basket design | The adaptive basket design includes various disease types that share a common biomarker. Patients with the same disease type are grouped into treatment cohorts called "baskets" [26]. These baskets can be expanded or stopped based on emerging trial evidence. The hypothesis behind this design is that the treatment works for a specific biomarker regardless of the disease type [25]. |
| Multi-arm multi-stage design | A design sequentially enrols participants at each interim analysis, multiple study treatments are tested based on the data obtained from all participants enrolled so far. Decisions can be made on whether to drop certain treatments that are less promising or select promising treatment(s) or stop the trial because sufficient evidence to reach a conclusion have been gathered (e.g., futility or efficacy). The least feature is treatment selection, so this shares similar properties with other AD such as adaptive treatment selection and adaptive seamless design. |
| Adaptive treatment selection | A type of MAMS design with options for selecting promising treatment(s) or dropping futile treatment(s) at an interim analysis as the main trial adaptation. If this was combined with other trial adaptations other than picking/dropping arms, then it was classed as a multiple adaptive design. |
|  |  |
| Adaptive seamless design | A design that aims to achieve objectives which were traditionally addressed in separate trial phases in one protocol, Decisions are made on whether to proceed to another trial phase based on interim results. The overall design type will change if multiple adaptations were planned. |
|  |  |
| Multiple adaptive design | If not stated otherwise as determined by the reviewer, this is an AD that incorporates multiple adaptations in a single trial, the combination could be either of the above-mentioned adaptive design [2], To avoid loss of information, the main objective of the design is also provided in Appendix 5 for trials classified as ‘multiple adaptive designs’. |

*In all these designs, changes to the design aspects are guided by pre-specified decision rules considering interim results.*

| **Targeted type of AD** | **Related terms commonly used in practice** |
| --- | --- |
| Group sequential design | “group sequential” OR “interim analysis” OR “interim analyses” |
| Multi-arm multi-stage design | “adaptive-platform” OR “multi-stage” OR “multi-arm multi-stage” OR “multiarm multistage” OR “multi-stage” |
| Adaptive platform design | “adaptive platform” |
| Adaptive treatment selection | “two stage” OR “pick the winner” OR “pick the loser” OR “drop the loser” OR “dose selection” OR “seamless” |
| Population enrichment design | Adaptive enrichment” OR “population enrichment” OR “patient enrichment” OR “enrichment design” OR “biomarker-adaptive” OR “biomarker-adaptive” OR “subpopulation” OR “subgroup selection” OR “subpopulation selection” |
| Response adaptive randomisation | “response adaptive” OR “response-adaptive” OR “adaptive randomisation” OR “adaptive randomisation” OR “outcome adaptive” OR “outcome-adaptive” |
| Adaptive hypothesis and sample size re-estimation design | “adaptive hypothesis” OR “adaptive hypotheses” |
| Sample size re-estimation design | “sample size adjustment” OR “sample size re-estimation” OR “sample size reestimation” OR “sample size modification” OR “sample size revision” OR “sample size reassessment” OR “sample size re-assessment” OR “sample size re-calculation” OR “sample size recalculation” OR “promising zone” |

**Appendix 2. Searching terms for the review.**

**Appendix 3. Specification for the data-extracting form**

| **Sort** | **Variable name** | **Description** |
| --- | --- | --- |
| 1 | NCTCODE | Registered No. |
| 2 | EXFL | Exclusion flag |
| 3 | EXRES | Reason for Exclusion (EX1=non-randomised trial, EX2=Study protocol or grant applications not written in the English language. EX3=Not an adaptive design in design’s nature) |
| 4 | EXRESORI | Detailed reason for exclusion |
| 5 | TRALSTA | Trial Status |
| 6 | STUTITL | Title |
| 7 | POSYEAR | Year of first posted (year) |
| 8 | STAYEAR | Year of the trial started (year) |
| 9 | SPOTYP | Type of the sponsor (public, industry, both) |
| 10 | DISORI | Condition or disease |
| 11 | DISCLASS | Systematic and organic classification of study disease (use the ICD-10 code) |
| 12 | GEOLOC | Geographic location of the study sites. (If one study was conducted in two or more continents, the detailed geographic location will be jointly displayed with "+") |
| 13 | RESPHA | Research phase (phase II/phase II/III/phase III/phase III/IV/phase IV/phase II/III/IV/not applicable) |
| 14 | ARMNUM1 | Numbers of treatment arms at the start of the trial (including the control arm) |
| 15 | ARMNUM2 | Number of arms after adaptations (only applicable for trials with adaptations on the number of arms 88=not stated,99=unfixed,00=concealed) |
| 16 | INT | Type of intervention(s) (drug/device/surgery/behavioural intervention, if more than one type of intervention then jointly displayed) |
| 17 | CONTTYP | Type of control (active/placebo/blank/standard of care) |
| 18 | ENDPRNUM | Number of primary endpoints |
| 19 | ENDDES | Description of the primary endpoint (detail every co-primary endpoint if applicable) |
| 20 | ENDCLS | Classification of primary endpoint(s) (binary, continuous, time-to-event, ordinal, if more than one type they jointly display with a "+") |
| 21 | HYPOCLS1 | Type of original primary hypothesis test(s) (superiority, non-inferiority, equivalence, etc.) |
| 22 | HYPOCLS2 | Type of primary hypothesis after adaptations (only applicable for trials with adaptations on hypothesis) |
| 23 | RANROYN | Was the randomisation ratio(s) stated (this should also be the initial randomisation ratio for response adaptive randomisation design trials)? |
| 24 | STADESIG | Statistical methods framework planned to be used for final analysis (Bayesian, frequentist, Bayesian and frequentist, not stated, concealed) |
| 25 | STAINTA | Statistical methods framework planned to be used for interim analysis (Bayesian, frequentist, Bayesian and frequentist, not stated, concealed) |
| 26 | SOFTDEA | How was the software used to design the trial described (only the software/software and package/macro/function/not stated) |
| 27 | SOFTSMPA | How was the software used for sample size calculation described (only the software/software and package/macro/function/not stated) |
| 28 | SOFTSTA | How was the software used for statistical analysis (interim and final) described (only the software/software and package/macro/function/not stated) |
| 29 | ADAPDES | Brief description of trial adaptation (early trial stopping option, dropping of futile arm, selecting of promising arm, not described, conceal) |
| 30 | ADAPT1 | Adaptation1 |
| 31 | ADAPT2 | Adaptation2 |
| 32 | ADAPT3 | Adaptation3 |
| 33 | ADAPT4 | Adaptation4 |
| 34 | ADAPT5 | Adaptation5 |
| 35 | STOPRES | Type of early stopping options considered for trial, treatment arm(s), or subpopulation(s) (efficacy, futility, efficacy or futility, not described, conceal) |
| 36 | STOCAT | Type of early stopping category (futility, efficacy, efficacy and futility, concealed) |
| 37 | ADTYPE | Type of adaptive design (The original description in the protocol) |
| 38 | ADCAT | Adaptive design category: designs with more than one predefined adaptation will be classified as 'multiple-adaptive design' |
| 39 | ADCAT_mul_gsd | Adaptive design category (group-sequential design, adaptive two stage design, adaptive treatment selection, adaptive Sample size re-estimation design, multi-arm multi-stage design, master protocol design, response adaptive randomisation, adaptive enrichment design, adaptive hypothesis design, adaptive seamless design, multiple adaptive design): only designs with GSD method plus additional adaptation will be deemed as multiple adaptive designs |
| 40 | BINYN | Was the futility boundary binding or non-binding, only applicable to group sequential design (binding, non-binding, not applicable) |
| 41 | INTNCO | Original records for number of interim analyses |
| 42 | INTAN | Number of interim looks, not include the final analysis (88=Unfixed,99=Not stated, 000=concealed) |
| 43 | INFO1ST | Information fraction at the first interim analysis (proportion, 88=Unfixed/flexible, 99=Not stated, 000=concealed. if the interim analysis was for more than one primary endpoints, then the information fraction will be based on the one triggered the interim analysis) |
| 44 | INF1STE | Information fraction at the first interim analysis for efficacy stopping (proportion, 88=Unfixed/flexible, 99=Not stated, 000=concealed. if the interim analysis was for more than one primary endpoints, then the information fraction will be based on the one triggered the interim analysis) |
| 45 | INF1STF | Information fraction at the first interim analysis for futility stopping (proportion, 88=Unfixed/flexible, 99=Not stated, 000=concealed. if the interim analysis was for more than one primary endpoints, then the information fraction will be based on the one triggered the interim analysis) |
| 46 | INFOFRA | Information fraction at each interim analysis, this should come from the study document. If the information fraction is not provided, it can be calculated by dividing the expected number of participants (or events) with available data at the planned interim analysis by the originally planned number of participants (or events).(vector, 88=Unfixed/flexible, 99=Not stated, 000=concealed) |
| 47 | MAMSYN | Was the trial designed in a multi-arm multi-stage manner? (refer to appendix 1,the definition of MAMS design) |
| 48 | DESDEYN | Are the interim decision-making criteria/rules described? (Yes, no, partially, concealed) |
| 49 | INTDES | If yes, what are the nature of interim decision rules? (Stopping rules, treatment selection rules, population selection rules, promising zone sample size re-estimation rules, response randomisation rules/algorithm etc.) |
| 50 | SAMPSTA | Statistical approach to sample size estimation (analytical, simulation, not described, concealed) |
| 51 | PAMSTAYN | Were the parameters for sample size calculated well stated? (Yes, no, partially, concealed) |
| 52 | PARJUSYN | Was the justification for sample size parameters described? (Yes, no, partially, concealed) |
| 53 | PARJUMIS | If no or just part of the justification for sample size parameters was described, then describe what is missing (effect size, nuisance parameters, factors related with adaptations, if more than one then jointly displayed with a ''+'') |
| 54 | PARREPYN | Were the sample size details adequately described to allow interpretation and reproducibility (yes, no, concealed)? |
| 55 | SAMPSUM | Brief summary of sample size estimation |
| 56 | SAFIXYN | Was the sample size for corresponding fixed design reported? (only applicable for group sequential design) (Yes, no, concealed, NA) |
| 57 | MENOTAS | Was the meaning of the targeted enrolment stated (yes, it is the minimum; yes, it is the maximum; yes, it is the corresponding fixed; yes, it is the expected; No) |
| 58 | SAMINYN | Was the minimum sample size reported? (Yes, no, concealed) |
| 59 | MINREP | How was the minimum sample size reported? (Stated directly in the protocol, inferred by the interim analysis plan and maximum sample size) |
| 60 | SAMAXYN | Was the maximum sample size reported? (Yes, no, concealed) |
| 61 | SAMEXYN | Was the expected sample size reported? (Yes, no, NA, concealed) |
| 62 | OPCYN | Other operating characteristics presented (yes, no, concealed) (If yes, answer the following questions) |
| 63 | OAIERR | Overall type I error (yes, no) |
| 64 | NOMSIGL | Nominal significance level (yes, no) |
| 65 | CUMPOW | Cumulative/stagewise power (yes, no) |
| 66 | GLOPOYN | Global power (yes, no) |
| 67 | EFFSPR | Early efficacy stopping probabilities (yes, no) |
| 68 | FUTLPR | Early futility stopping probabilities (yes, no) |
| 69 | SAMRPR | Probabilities for adaptive sample size re-estimating (yes, no) |
| 70 | OTHADPR | Probabilities for other adaptations (adding/dropping arms, hypothesis switching) (yes, no) |
| 71 | SAMREYN | Is there an adaptation about sample size re-estimation (yes, no, concealed) (If yes answer the following 3 questions) |
| 72 | BLINSTA | Estimated under blind or Unblind situation |
| 73 | COMPSTA | Comparative or non-comparative |
| 74 | COMBAS | Based on nuisance parameters or interim treatment effect or both |
| 75 | SAMPPLA | Planned sample size (for time-to-event endpoints, this should be the number of events) |
| 76 | SAMPACT | Actual sample size (number of events) when trial was completed (only applicable for completed trials) |
| 77 | STOACT | Stopping type (efficacy, futility, reaching the final stage) |
| 78 | SAMDRES | Reason for sample size drift (early stopping, sample size re-estimation, additional arm, population enrichment) |

**Appendix 4 Cross-validation history**

| Base variable | **Compared variable** | **Objectives** | **Modification history** |
| --- | --- | --- | --- |
| COMPSTA | COMBAS | to check the comparative status against the type of sample size re-estimating method | 3 cases of comparative sample size re-estimation+ sample size re-estimated on nuisance parameters were disclosed, corrections were made after double check 1 case of non-comparative sample size re-estimation+ sample size re-estimated on interim effect and nuisance' was found, correction was made accordingly. |
| SPOTYP | RESPHA | to check the type of sponsor against the research phase | 1 case with phase classed as "non-applicable" was recorded as sponsored by industry incorrectly, correction was made after double check |
| PAMSTAYN | PARJUSYN | to check the consistency between the stating and justifying the parameters for sample size calculation | 2 cases were corrected after double check |
| ARMNUM1 | ADCAT | to check the consistency between number of arms and type of adaptive design | 3 cases were corrected since the misclassification of ‘group-sequential design’ when more than two arms were tested |
| STOCAT | EFFSPR, FUTLPR | logic check on the type of early stopping | 4 corrections were made respectively |

**Appendix 5. Further information on the classification of adaptive design.**

| **Features related to AD** | **Type of the sponsor**  **N (%)** | | **Total**  **(n=265)** |
| --- | --- | --- | --- |
|  | **Industry**  **(n=164)** | **Public sector**  **(n=101)** |  |
| Type of AD |  |  |  |
| Group sequential design | 98 (59.8) | 71 (70.3) | 169 (63.8) |
| Multiple adaptive design | 41 (25.0) | 20 (19.8) | 61 (23.0) |
| Sample size re-estimation design | 10 (6.1) | 3 (3.0) | 13 (4.9) |
| Adaptive treatment selection | 3 (1.8) | 2 (2.0) | 5 (1.9) |
| Adaptive seamless design | 3 (1.8) | 1 (1.0) | 4 (1.5) |
| Adaptive basket design | 0 (0) | 1 (1.0) | 1 (0.4) |
| Adaptive enrichment design | 0 (0) | 1 (1.0) | 1 (0.4) |
| Adaptive hypothesis design | 1 (0.6) | 0 (0) | 1 (0.4) |
| Response adaptive randomisation | 0 (0) | 1 (1.0) | 1 (0.4) |
| Concealed | 8 (4.9) | 1 (1.0) | 9 (3.4) |
|  |  |  |  |
| Main class when multiple adaptations were designed ^a^ | (n= 41) | (n= 20) | (n= 61) |
| Sample size re-estimation design | 26 (63.4) | 8 (40.0) | 35 (57.4) |
| Adaptive treatment selection | 8 (19.5) | 5 (25.0) | 12 (19.7) |
| Adaptive enrichment design | 4 (9.8) | 1 (5.0) | 5 (8.2) |
| Adaptive platform design | 0 (0) | 4 (20.0) | 4 (6.6) |
| Response adaptive randomisation | 1 (2.4) | 2 (10.0) | 3 (4.9) |
| Adaptive seamless design | 2 (4.8) | 0 (0) | 2 (3.2) |

*^a^ This was the main objective out of multiple adaptations’ combining, based on reviewer’s judgement.*

**Appendix 6. Replicability of sample size calculation across types of adaptive design.**

| **Type of adaptive design** | **Replicability** | | |
| --- | --- | --- | --- |
|  | **YES** | **NO** | **Concealed** ^a^ |
| Group-sequential design | 147 (87.0) | 18 (10.7) | 4 (2.4) |
| Multiple adaptive designs | 48 (78.7) | 13 (21.3) | 0 (0) |
| Sample size re-estimation design | 11 (84.6) | 2 (15.4) | 0 (0) |
| Adaptive treatment selection | 3 (60.0) | 2 (40.0) | 0 (0) |
| Adaptive seamless design | 4 (100) | 0 (0) | 0 (0) |
| Adaptive basket design | 1 (100) | 0 (0) | 0 (0) |
| Adaptive enrichment design | 1 (100) | 0 (0) | 0 (0) |
| Adaptive hypothesis design | 1 (100) | 0 (0) | 0 (0) |
| Response adaptive randomisation | 0 (0) | 1 (100) | 0 (0) |
| Concealed | 0 (0) | 1 (11.1) | 8 (88.9) |

*^a^ This means the sample size calculation part was concealed due to confidentiality considerations, so the replicability cannot be determined.*
